# Diabetic Retinopathy in Bahraini Primary Care: A Cross-Sectional Study of Prevalence and Clinical Predictors

**DOI:** 10.7759/cureus.109530

**Published:** 2026-05-23

**Authors:** Mahmood Alawainati, Nora AlGhareeb, Reem Alhouli, Mariam Buhejji, Haya F Mohammad, Hessa AlBuainain, Lateefa Rashed Daraj, Alaa Alqallaf, Aysha Almulla, Wahaj Alenezi

**Affiliations:** 1 Medicine, Royal College of Surgeons in Ireland - Bahrain, Manama, BHR; 2 Family Medicine, Primary Healthcare Centers, Manama, BHR; 3 Medicine, Arabian Gulf University, Manama, BHR; 4 Internal Medicine, Salmaniya Medical Complex, Manama, BHR; 5 General Practice, Bahrain Defense Force Hospital, Riffa, BHR; 6 Medicine, Bahrain Oncology Center, Manama, BHR

**Keywords:** diabetes mellitus, diabetic retinopathy, noncommunicable diseases, ophthalmology, retinal diseases

## Abstract

Introduction

Diabetic retinopathy (DR) remains a prevalent cause of preventable blindness and one of the most serious complications of diabetes mellitus globally, which can be minimized by periodic screening and risk factor management. Nonetheless, few studies have assessed its prevalence and predictors among diabetic patients in primary care settings. This study aimed to determine the prevalence and predictors of DR among diabetic patients in primary care in Bahrain.

Methods

A cross-sectional study was conducted in October-November 2025 across all primary healthcare centers in Bahrain. All adult patients with diabetes attending diabetic clinics were included. DR was assessed by a specialist optometrist using a non-mydriatic retinal camera (TOPCON NW500). Retina assessment results included normal retina, non-proliferative retinopathy and proliferative retinopathy. Sociodemographic characteristics, comorbidities, and risk factors were collected. Descriptive and inferential analyses were done. A P-value <0.05 was considered statistically significant.

Results

A total of 594 patients were included, with a median age of 61 years. Most participants were male (337, 56.7%), Bahraini (506, 85.2%), and had type 2 diabetes (575, 96.8%). Dyslipidemia (463, 77.9%) and hypertension (397, 66.8%) were the commonest comorbidities. DR was noted in 88 (14.8%) patients; 13% (n=77) had non-proliferative DR and 1.8% (n=11) had proliferative DR. Univariate analysis showed that patients with retinopathy were older (P<0.001), had a longer diabetes duration (P<0.001), and higher rates of hypertension (P=0.001), dyslipidemia (P=0.009), chronic kidney disease (P<0.001), and ischemic heart disease (P=0.042). Additionally, higher glycated hemoglobin (P=0.014), and triglycerides (P=0.022), and lower glomerular filtration rate (P<0.001) were found among patients with retinopathy. Logistic regression identified a longer diabetes duration (OR=1.576, P<0.001), hypertension (OR=1.912, P=0.039), abnormal monofilament test (OR=2.92, P=0.027), and higher HbA1c (OR=1.013, P=0.033) as predictors of DR.

Conclusion

DR affects one in seven diabetic patients attending primary care in Bahrain, particularly those with a longer disease duration, hypertension, poor glycemic control, and neuropathy. Annual retinal screening, optimal glycemic control, and risk factor management by primary care professionals are essential to reduce its sequelae.

## Introduction

Diabetes mellitus (DM) is a chronic disease that affects millions of people worldwide [1]. In 2020, it was estimated that more than 450 million individuals were living with diabetes globally, and this number is projected to increase to approximately 700 million by 2045 [2]. In the Gulf Cooperation Council (GCC) countries, the prevalence of diabetes is among the highest in the world, with a range between 8% and 22% [3]. In Bahrain, a national survey reported that nearly 15% of the population is affected by type 2 DM [4].

Patients with diabetes are at an increased risk of developing multisystem complications, including macrovascular complications such as ischemic heart disease, cerebrovascular disease, and peripheral vascular disease, as well as microvascular complications such as diabetic nephropathy, neuropathy, and retinopathy [5]. Diabetic retinopathy (DR) is a leading cause of visual impairment and vision loss in adults and is associated with increased morbidity [6]. The pathogenesis of DR is multifactorial and complex. Chronic hyperglycemia activates several metabolic pathways that lead to vascular changes, including retinal blood vessel dilation, altered blood flow, and pericyte loss. Hyperglycemia also results in an inflammatory response that damages retinal endothelial cells and the blood-retina barrier. With time, these changes result in vascular leakage, retinal ischemia, neovascularization, and bleeding [7,8].

Several studies were conducted to determine the prevalence of DR across the world. A systematic review conducted across GCC countries, including 20 studies and 61,855 diabetic patients from 2003 to 2019, found an overall DR prevalence of 20.5%, with the highest rate in Saudi Arabia (69.8%) and the lowest rate in the UAE (6.0%) [9]. A cross-sectional study of more than 10,000 patients conducted in Alexandria and the North-West Delta region estimated the prevalence of DR to be 32.49% [10]. The study also found that high random blood glucose and longer diabetes duration were significantly associated with increased retinopathy risk. A systematic review of 59 studies revealed that the pooled prevalence of DR was 22.27%, with more than 100 million patients suffering from the disease. Surprisingly, the study found that the highest rate of DR was seen in Africa (35.90%) and the lowest in South and Central America (13.37%) [11].

Additionally, some studies were conducted to determine the risk factors and predictors of DR. Advanced age, obesity, hypertension, uncontrolled diabetes, insulin use, smoking, and prolonged diabetes were predictors of DR [12,13]. Additionally, patients with low levels of low-density lipoprotein cholesterol, anemia, and poor diabetes control had higher rates of DR [14].

Studying the prevalence and associated factors of DR in Bahrain is important given the high prevalence of diabetes in Bahrain and the significant impact of DR on visual impairment and blindness among adults. While several regional and international studies have examined DR, data specific to Bahrain remain limited. Furthermore, while most global literature focuses on hospital-based populations, there is a lack of evidence regarding the efficacy of decentralized, nurse-led screening using non-mydriatic imaging in the GCC region. This study provides recent, community-based data about the prevalence of DR and its associated risk factors within Bahrain, thereby addressing an important gap in the literature. Moreover, understanding the prevalence and risk factors of DR among individuals with diabetes is essential for early detection and effective prevention aimed at reducing diabetes-related visual complications.

## Materials and methods

A cross-sectional study was conducted between October and November 2025 in primary healthcare centers in Bahrain. The primary healthcare system in Bahrain includes 26 centers distributed in four governorates. All primary healthcare centers have non-communicable disease clinics, which provide medical services to patients with type 2 DM, multidisciplinary teams comprising family physicians and diabetes specialist nurses. Ethical approval for the study was granted by the Ethics Committee of Primary Healthcare in Bahrain.

Adults (aged 18 years and above) with DM attending non-communicable disease clinics in Bahrain were included in the present study. Patients with emergency conditions, severe cognitive impairment, and communication difficulties preventing participation were excluded.

Based on an assumed 50% prevalence of retinopathy among type 2 diabetic patients [9], a 5% precision, and a 95% confidence interval, the calculated minimum sample size was 378 patients. The sample size (N) was calculated using the following equation. N = Z² × p(1 − p) / d² = (1.96)² × 0.5(1 − 0.5) / (0.05)² = 384, where n = required sample size, Z = 1.96 for 95% confidence interval, p = assumed prevalence (50%), and d = precision (5%). To increase the power, a sample of more than 500 was targeted. To ensure a representative sample, two months were randomly selected via a computer-generated sequence to include all consecutive patients attending non-communicable disease clinics during that period.

A structured data collection tool was made to collect the data. It included three parts; the first part assessed baseline characteristics of the participants including age, sex, nationality, education, marital status, comorbidities, medications, and duration of diabetes, the second part assessed lifestyle factors such as smoking, and alcohol use, the third part assessed the examination findings and the fourth part assessed laboratory findings including glycated hemoglobin (HbA1c), fasting plasma glucose, and lipid profile. Additionally, the prescribed medications were noted. The biochemical markers and the retinal examination were assessed simultaneously.

Retina examination was performed by a specialist ophthalmology nurse (senior optometrist) who obtained special training in retina screening and had a Bachelor's in Optometry. A non-mydriatic retinal camera (TOPCON NW500, Topcon Healthcare, Tokyo, Japan) that provides a 50° field of view and supports stereoscopic, bilateral two-field retinal photography was used to assess the retina. This device allows for imaging through pupils as small as 2.0 mm, reducing the need for pharmacological dilation and improving patient throughput in a primary care setting. Retinal assessment results included normal retina, non-proliferative retinopathy, and proliferative retinopathy. Neuropathy was assessed through neuropathic symptoms and the monofilament test; renal complications were assessed through laboratory tests, and diabetes control was assessed through HbA1c and fasting plasma glucose. DM was diagnosed according to the American Diabetes Association criteria, and the duration of diabetes was calculated from the time of diagnosis to the data collection period. Glycemic control was assessed using HbA1c levels, with <7% indicating controlled diabetes, while 7% or more indicating uncontrolled diabetes.

Data was entered in IBM SPSS Statistics for Windows, Version 25 (Released 2017; IBM Corp., Armonk, New York, United States). Then, data cleaning and testing for data normality were done. Categorical variables were presented as frequencies and percentages, while continuous variables were summarized as medians with interquartile ranges. The Mann-Whitney U test was used for continuous variables, while Fisher’s exact and chi-square tests were used to compare categorical data. Logistic regression was done, and the final step of the analysis was reported. Inter-observer and intra-observer reliability were assessed using the κ coefficient. Collinearity between age and duration of diabetes was tested and found to be non-significant (Variance Inflation Factor= 1.135 and Tolerance= 0.881). A P-value of <0.05 was considered statistically significant.

## Results

A total of 594 patients were included in the study, with a median age of 61 ± 13 years (response rate= 93.5%). Most participants were male (337, 56.7%), Bahraini (506, 85.2%) and had type 2 DM (575, 96.8%), with a median duration of diabetes of 11 years. Additionally, 14% (n=83) of the patients were current smokers, and 2.2% (n=13) were alcohol consumers. Hypertension (397, 66.8%) and dyslipidemia (463, 77.9%) were the most common comorbidities. In terms of treatment, metformin (462, 77.8%), sulphonylurea (258, 43.4%), and dipeptidyl peptidase-4 inhibitors (255, 42.9%) were the most prevalent diabetes medications, while statins (480, 80.8%), angiotensin-converting enzyme inhibitors, angiotensin II receptor blockers (262, 44.1%), and aspirin (182, 30.6%) were the most prevalent non-diabetic medications. Additionally, 24.2% (n=144) of patients reported sensory symptoms, 4.5% (n=27) had abnormal monofilament test results, and 2.5% (n=15) had abnormal Doppler findings (Table 1).

**Table 1:** Sociodemographic, Clinical Characteristics, and Treatments of the Study Population (TN=594) DPP-4: Dipeptidyl peptidase 4; GLP-1: glucagon-like peptide 1; SGLT-2: sodium-glucose co-transporter 2; ACEI: angiotensin-converting enzyme inhibitor; ARB: angiotensin II receptor blocker

Variable		N (%)
Age, median ± IQR		61.00 ±13
Sex	Male	337 (56.7)
	Female	257 (43.3)
Nationality	Bahraini	506 (85.2)
	Non-Bahraini	88 (14.8)
Smoking	Yes	83 (14)
	No	462 (77.8)
	Ex-smoker	49 (8.2)
Alcohol	Yes	13 (2.2)
	No	574 (96.6)
	Ex-drinker	7 (1.2)
Type of diabetes	1	19 (3.2)
	2	575 (96.8)
Duration in years, median ±IQR		11.00 ±13
Diabetes treatments	Metformin	462 (77.8)
	Sulphonylurea	258 (43.4)
	DPP-4 inhibitors	255 (42.9)
	Insulin	223 (37.5)
	SGLT-2 inhibitors	54 (9.1)
	GLP-1 agonists	28 (4.7)
Other medications	Statins	480 (80.8)
	ACEI/ARBs	262 (44.1)
	CCB	125 (21)
	Aspirin	182 (30.6)
	Diuretic	75 (12.6)
	Clopidogrel	7 (1.2)
	Beta blockers	44 (7.4)
Comorbidities	Hypertension	397 (66.8)
	Dyslipidemia	463 (77.9)
	Ischemic Heart Disease	59 (9.9)
	Sensory: burning, numbness, tingling	144 (24.2)
	Monofilament Test	27 (4.5)
	Doppler Ultrasound (Arterial flow) /	15 (2.5)
	Diabetic Foot	32 (5.4)

**Figure 1:**
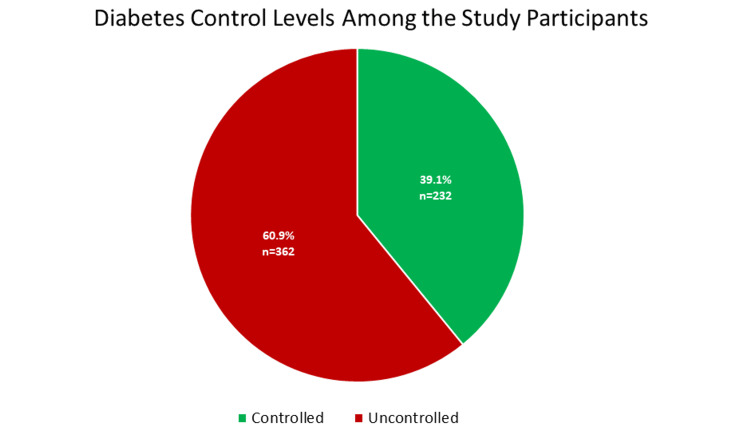
Glycemic Control Status Among the Study Participants

Out of the total sample, 39.1% (n=232) had controlled diabetes, while the majority (362, 60.9%) had uncontrolled diabetes (Figure 1).

**Figure 2:**
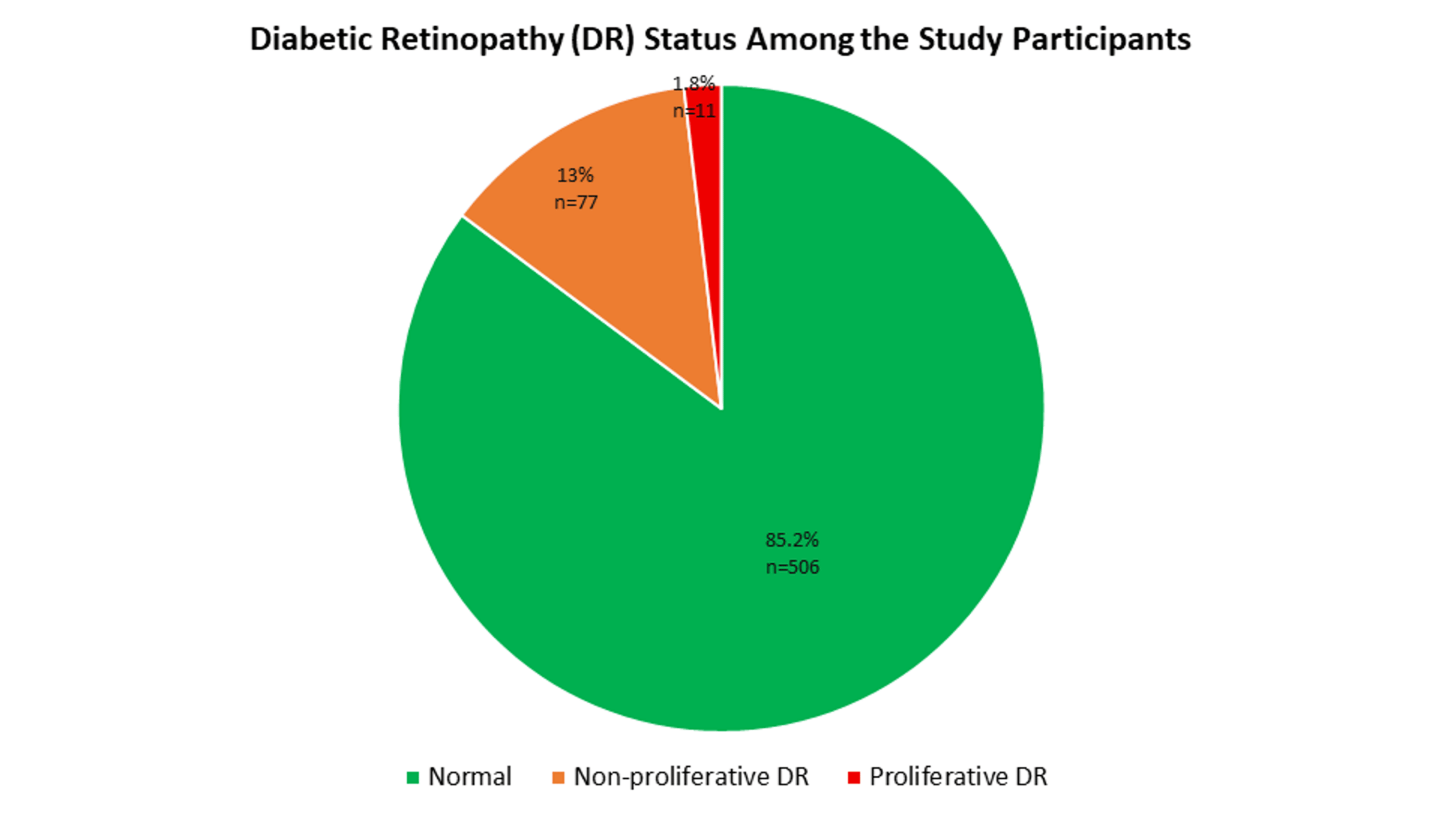
Diabetic Retinopathy Status Among the Study Participants DR: Diabetic retinopathy

Regarding retinopathy, 85.2% (n=506) of patients had normal retina, whereas 14.8% (n=88) (95% CI: 11.9%-17.7%) had retinopathy. Non-proliferative DR affected 13% (n=77) of patients while proliferative DR affected 1.8% (n=11) of the patients with DR (Figure 2). High levels of inter-observer (κ = 0.78, 95% CI: 0.71-0.84) and intra-observer reliability (κ = 0.85, 95% CI: 0.79-0.90) were noted.

Table 2 showed that patients with retinopathy were significantly older (P<0.001) and had a longer duration of diabetes (P<0.001) compared to their counterparts. Patients with hypertension (P=0.001), dyslipidemia (P=0.009), chronic kidney disease (P<0.001), and ischemic heart disease (P=0.042) had higher rates of retinopathy. Additionally, retinopathy was also more prevalent among those with sensory neuropathic symptoms (P=0.020), abnormal monofilament results (P<0.001), and abnormal doppler findings (P<0.001).

**Table 2:** Comparison of Sociodemographic and Clinical Characteristics Between Patients with Retinopathy and Patients with Normal Retina

		Normal Retina, N=506	Retinopathy, N=88	P-value
Age, median ± IQR		61.00 ±15.00	65.00 ±13.00	<0.001
Sex	Male	289(85.8)	48(14.2)	0.653
	Female	217(84.4)	40(15.6)	
Nationality	Bahraini	430(85)	76(15)	0.736
	Non-Bahraini	76(86.4)	12(13.6)	
Smoking	Yes	72(86.7)	11(13.3)	0.721
	No	394(85.3)	68(14.7)	
	Ex-smoker	40(81.6)	9(18.4)	
Alcohol	Yes	10(76.9)	3(23.1)	0.698
	No	490(85.4)	84(14.6)	
	Ex-drinker	6(85.7)	1(14.3)	
Type of diabetes	1	19(100)	0(0)	0.093
	2	487(84.7)	88(15.3)	
Duration in years, median ± IQR		10.00 ±12.00	20.00 ±8.00	<0.001
Hypertension	Yes	325(81.9)	72(18.1)	0.001
	No	181(91.9)	16(8.1)	
Dyslipidemia	Yes	385(83.2)	78(16.8)	0.009
	No	121(92.4)	10(7.6)	
Chronic Kidney Disease based on GFR	Yes	54(70.1)	23(29.9)	<0.001
	No	452(87.4)	65(12.6)	
Ischemic Heart Disease	Yes	45(76.3)	14(23.7)	0.042
	No	461(86.2)	74(13.8)	
Sensory: burning, numbness, tingling	Yes	114(79.2)	30(20.8)	0.020
	No	392(87.1)	58(12.9)	
Monofilament Test	Normal	491(86.6)	76(13.4)	<0.001
	Abnormal	15(55.6)	12(44.4)	
Doppler Ultrasound (Arterial flow)	Normal	499(86.2)	80(13.8)	<0.001
	Abnormal	7(46.7)	8(53.3)	
DM control (A1C level)	<7%	204(87.9)	28(12.1)	0.132
	7% or more	302(83.4)	60(16.6)	

Biochemical markers showed that patients with retinopathy had significantly higher HbA1c levels (63 vs. 56 mmol/mol, P=0.014), higher triglycerides (1.70 vs. 1.50 mmol/L, P=0.022), and lower eGFR (80.5 vs. 96 mL/min/1.73m², P<0.001) compared to those without retinopathy (Table 3).

**Table 3:** Comparison of Laboratory Markers Between Patients with Normal Retina and Retinopathy

	Overall	Normal Retina, N=506	Retinopathy, N=88	P-value
Fasting Blood Glucose (mmol/L), median± IQR	7.20±3.40	7.20±3.00	7.35±4.30	0.482
Hemoglobin A1C (HbA1c) (mmol/mol), median± IQR	57.00±25.50	56.00±23.00	63.00±27.00	0.014
Total Cholesterol in mmol/L, median± IQR	4.00±1.20	4.00±1.20	4.10±1.30	0.741
LDL in mmol/L, median± IQR	2.13±1.07	2.14±1.07	2.09±1.05	0.275
HDL in mmol/L, median± IQR	1.07±0.36	1.08±0.36	1.07±0.35	0.296
Triglycerides (mmol/L, median± IQR	1.55±1.00	1.50±1.00	1.70±1.10	0.022
Vitamin B12 in pmol/L, median± IQR	253.00±115.30	250.30±112.4	267.00±132.7	0.108
TSH in mI U/L, median± IQR	1.96±1.52	1.93±1.54	2.01±1.41	0.495
Sodium in mmol/L, median± IQR	139.0±3.0	139.00±3.00	139.00±4.00	0.692
eGFR in mL/min/1,73m^2, median± IQR	94.00±41.0	96.00±34.00	80.50±50.00	<0.001

As shown in Table 4, logistic regression analysis revealed that a longer duration of diabetes (OR=1.576, P<0.001), presence of hypertension (OR=1.912, P=0.039), abnormal monofilament test (OR=2.92, P=0.027), and higher HbA1c (OR=1.013, P=0.033) were independent associated factors of DR.

**Table 4:** Final Step of Logistic Regression Analysis of Factors Associated with Retinopathy Variables entered on step 1: Age, Duration in years, Hypertension, Dyslipidemia, Chronic Kidney Disease, Ischemic Heart Disease, Sensory: burning, numbness, tingling, Monofilament Test, Doppler Ultrasound (Arterial flow)/Palpation, Hemoglobin A1C (HbA1c, %) (mmol/mol), Triglycerides (mmol/L)

Variable	Odds ratio (95% Confidence Interval)	P-value
Diabetes Duration	1.576(1.446-1.607)	<0.001
Hypertension	1.912(1.035-3.533)	0.039
Dyslipidemia	1.891(0.890-4.016)	0.097
Monofilament Test	2.92 (1.13–7.58)	0.027
Doppler Ultrasound (Arterial flow)/Palpation	0.358 (0.107-1.197)	0.095
Hemoglobin A1C (HbA1c, %) (mmol/mol)	1.013 (1.001-1.026)	0.033

## Discussion

This study aimed to determine the prevalence and associated factors of DR among patients with diabetes mellitus attending non-communicable disease clinics in primary healthcare centers in Bahrain. The results showed that the prevalence of DR was 14.8%, affecting nearly one in seven diabetic patients. The study also found that prolonged diabetes duration, hypertension, abnormal monofilament test, and high A1C were associated factors of DR.

Compared to other studies, the present study reported a relatively lower rate of DR among patients with diabetes. For example, other GCC countries reported a pooled prevalence of 20.5% [9]. A higher rate reaching to 22.3% and 32.5% were reported in Africa and Egypt, respectively. The relatively low prevalence seen in our study could be attributed to the presence of structured screening programs in primary care in Bahrain and high levels of awareness among patients, as evident from a previous study, which showed adequate levels of knowledge and practices about foot care among diabetic patients in Bahrain [15]. Bahrain's established NCD clinic infrastructure could help in facilitating earlier intervention compared to hospital-based studies. Furthermore, our study demonstrates the efficacy of a task-shifting model where trained optometry nurses utilize automated retinal imaging. This decentralized approach can serve as a blueprint for other high-prevalence regions facing a shortage of ophthalmologists. Additionally, the low prevalence of DR may be explained by the fact that the study population included patients attending specialized diabetic clinics in primary care, who may be more engaged with healthcare and better managed than those attending general clinics. Furthermore, the use of a binary classification (presence/absence) of DR may have underestimated milder or early-stage cases. Nonetheless, the observed prevalence of DR in this study represents a significant burden on the healthcare system due to the high risk of impairment associated with DR.

The present study found that prolonged diabetes duration was an associated factor of DR. Prolonged and uncontrolled hyperglycemia can lead to microvascular damage in the retina, therefore increasing the prevalence and progression of retinopathy. Similar findings were also reported in the literature. Interestingly, some studies found that diabetes duration was the strongest risk factor for DR [12,13,16,17]. With a prolonged history of diabetes, further damage to the retinal blood vessels occurs, which increases the risk of developing or worsening DR. This is further supported by the significant association between uncontrolled A1C levels and the higher risk of DR, as noted in this study as well as previous studies [12,18,19].

Furthermore, hypertension was found to be an associated factor of DR in this study. Hypertension is known to increase retinopathy risk and worsen its outcomes. Studies found that even slightly high blood pressure readings >120/80 mmHg increased the risk of DR [20,21]. Hypertension itself was found to lead to several eye diseases, which are often exacerbated when diabetes is also present and vice versa [12,22]. Therefore, controlling hypertension is beneficial in reducing the incidence and burden of DR among patients with diabetes mellitus [23,24].

Moreover, the presence of neuropathy as detected by the abnormal monofilament test was also a risk factor for DR. This reflects a shared underlying microvascular pathophysiology that is affected by hyperglycemia and vascular impairment. Similar findings were found in the literature as well [25]. Clinically, neuropathic symptoms and signs may be used as an indicator of concurrent retinal disease.

Moreover, although participant age was significantly associated with DR in univariate analysis, logistic regression did not identify age as an independent associated factor of DR. Age is a common risk factor for several eye diseases, and might increase the risk of DR as noted in some studies [26]. This study found no association between cholesterol levels, smoking, and DR. Nonetheless, such associations have been reported in the literature [12-14]. 

The study highlighted the importance of routine retinal screening programs, especially for patients with longer diabetes duration, hypertension, or poor glycemic control. In addition, the results also emphasize the importance of integrated chronic disease management that includes tight glycemic and blood pressure control in patients with DM to prevent long-term complications including DR.

This study has several strengths. First of all, all primary care centers were included, and a probability sampling was used, which reduced the probability of selection bias and increased generalizability. Secondly, the relatively large sample size further strengthens the reliability of the findings. Thirdly, the study is one of the few studies that were done to evaluate DR prevalence and associated factors in the primary care setting in Bahrain. However, the study has some limitations as well. Patients attending specialized clinics were included only. This might affect the actual prevalence of DR, as patients not attending these clinics might have a higher prevalence of DR. Additionally, some important variables, such as socioeconomic status, diet, physical activity, and medication adherence, were not assessed, which could also influence retinopathy risk.

## Conclusions

In conclusion, this study revealed that DR affected nearly one in seven diabetic patients, with higher rates among patients with a longer duration of diabetes, hypertension, poor glycemic control, and peripheral neuropathy. Primary care physicians should ensure adequate glycemic control and blood pressure levels to minimize the rate and burden of DR. Regular and early retinal assessment is essential for patients with diabetes, especially those with poor glycemic control, long-standing disease, or hypertension, to reduce the risk and progression of DR. These findings support the integration of retinal screening within primary care protocols to mitigate vision loss in patients with DM. Further studies are needed to confirm causal relationships of retinopathy risk factors and assess the impact of sociodemographic status, diet, and physical activity. 

## References

[1] Hossain MJ, Al-Mamun M, Islam MR (2024). Diabetes mellitus, the fastest growing global public health concern: early detection should be focused. Health Sci Rep.

[2] Saeedi P, Petersohn I, Salpea P (2019). Global and regional diabetes prevalence estimates for 2019 and projections for 2030 and 2045: results from the International Diabetes Federation Diabetes Atlas, 9(th) edition. Diabetes Res Clin Pract.

[3] Aljulifi MZ (2021). Prevalence and reasons of increased type 2 diabetes in Gulf Cooperation Council Countries. Saudi Med J.

[4] (2018). Bahrain National Health Survey 2018. Bahrain National Health Survey.

[5] Khunti K, Chudasama YV, Gregg EW (2023). Diabetes and multiple long-term conditions: a review of our current global health challenge. Diabetes Care.

[6] Kropp M, Golubnitschaja O, Mazurakova A (2023). Diabetic retinopathy as the leading cause of blindness and early predictor of cascading complications-risks and mitigation. EPMA J.

[7] Wang W, Lo AC (2018). Diabetic retinopathy: pathophysiology and treatments. Int J Mol Sci.

[8] Richardson E, Farrell R (2019). Diabetic retinopathy in youth-onset type 2 diabetes mellitus. Pediatric Type II Diabetes.

[9] Mohamed Z, Al-Natour M, Al Rahbi H (2024). Prevalence of diabetic retinopathy among individuals with diabetes in gulf cooperation council countries: a systematic review and  meta-analysis. Oman Med J.

[10] Elmassry A, Ahmed IS, Adly N, Torki M (2023). Prevalence of diabetic retinopathy in patients with diabetes in Alexandria and North-West Delta, Egypt. Int Ophthalmol.

[11] Teo ZL, Tham YC, Yu M (2021). Global prevalence of diabetic retinopathy and projection of burden through 2045: systematic review and meta-analysis. Ophthalmology.

[12] Ghamdi AH (2020). Clinical predictors of diabetic retinopathy progression; a systematic review. Curr Diabetes Rev.

[13] Roto A, Farah R, Al-Imam M, Q Al-Sabbagh M, Abu-Yaghi N (2022). Prevalence, characteristics and risk factors of diabetic retinopathy in type 2 diabetes mellitus patients in Jordan: a cross-sectional study. J Int Med Res.

[14] Boonsaen T, Choksakunwong S, Lertwattanarak R (2021). Prevalence of and factors associated with diabetic retinopathy in patients with diabetes mellitus at Siriraj hospital - Thailand's largest national tertiary referral center. Diabetes Metab Syndr Obes.

[15] Habbash F, Saeed A, Abbas F, Ajlan BY, Abdulla F, Al-Sayyad AS (2019). Knowledge and practice regarding foot care in patients with diabetes mellitus attending diabetic clinics in health centers in the Kingdom of Bahrain. Int J Med Public Health.

[16] Zhang D, Zhang Y, Kang J, Li X (2024). Nonlinear relationship between diabetes mellitus duration and diabetic retinopathy. Sci Rep.

[17] Jerneld B, Algvere P (1986). Relationship of duration and onset of diabetes to prevalence of diabetic retinopathy. Am J Ophthalmol.

[18] (2021). Development and progression of diabetic retinopathy in adolescents and young adults with type 2 diabetes: results from the TODAY Study. Diabetes Care.

[19] Morya AK, Ramesh PV, Nishant P, Kaur K, Gurnani B, Heda A, Salodia S (2024). Diabetic retinopathy: a review on its pathophysiology and novel treatment modalities. World J Methodol.

[20] Li YT, Wang Y, Hu XJ (2021). Association between systolic blood pressure and diabetic retinopathy in both hypertensive and normotensive patients with type 2 diabetes: risk factors and healthcare implications. Healthcare (Basel).

[21] Zhang M, Wu J, Wang Y, Wu J, Hu W, Jia H, Sun X (2023). Associations between blood pressure levels and diabetic retinopathy in patients with diabetes mellitus: a population-based study. Heliyon.

[22] Dziedziak J, Zaleska-Żmijewska A, Szaflik JP, Cudnoch-Jędrzejewska A (2022). Impact of arterial hypertension on the eye: a review of the pathogenesis, diagnostic methods, and treatment of hypertensive retinopathy. Med Sci Monit.

[23] (2025). 12. Retinopathy, neuropathy, and foot care: standards of care in diabetes-2025. Diabetes Care.

[24] Do DV, Han G, Abariga SA, Sleilati G, Vedula SS, Hawkins BS (2023). Blood pressure control for diabetic retinopathy. Cochrane Database Syst Rev.

[25] Rasheed R, Pillai GS, Kumar H, Shajan AT, Radhakrishnan N, Ravindran GC (2021). Relationship between diabetic retinopathy and diabetic peripheral neuropathy - neurodegenerative and microvascular changes. Indian J Ophthalmol.

[26] Li Q, Wang M, Li X, Shao Y (2023). Aging and diabetic retinopathy: inherently intertwined pathophysiological processes. Exp Gerontol.

